# Drug Repurposing: Exploring Potential Anti-Cancer Strategies by Targeting Cancer Signalling Pathways

**DOI:** 10.3390/biology13060386

**Published:** 2024-05-28

**Authors:** Natalia Haddad, Sara Magura Gamaethige, Nadine Wehida, Ahmed Elbediwy

**Affiliations:** Department of Biomolecular Sciences, Kingston University London, Kingston-upon-Thames KT1 2EE, UK

**Keywords:** drug repurposing, cancer therapy, hippo signalling pathways, Wnt signalling, immunotherapy, anti-cancer, anti-tumour, cancer signalling, cancer targeting, drug design

## Abstract

**Simple Summary:**

The repurposing of drugs is an important and rapidly growing field in the treatment of cancer. The main advantage of drug repurposing is using currently established drugs for various diseases and testing them on newer diseases, such as cancer. This strategy allows drug developers to reduce the time for a drug to be made available after clinical trials and testing by using drugs that have already gone through the process. The key to its use is to target the intricate signalling pathways in cancer, which contribute to the initiation, progression, and severity of cancer. Such pathways, if targeted correctly using repurposed drugs, could result in the regression of the tumour or, in some cases, a potential curative effect.

**Abstract:**

The repurposing of previously clinically approved drugs as an alternative therapeutic approach to treating disease has gained significant attention in recent years. A multitude of studies have demonstrated various and successful therapeutic interventions with these drugs in a wide range of neoplastic diseases, including multiple myeloma, leukaemia, glioblastoma, and colon cancer. Drug repurposing has been widely encouraged due to the known efficacy, safety, and convenience of already established drugs, allowing the bypass of the long and difficult road of lead optimization and drug development. Repurposing drugs in cancer therapy is an exciting prospect due to the ability of these drugs to successfully target cancer-associated genes, often dysregulated in oncogenic signalling pathways, amongst which are the classical cancer signalling pathways; WNT (wingless-related integration type) and Hippo signalling. These pathways play a fundamental role in controlling organ size, tissue homeostasis, cell proliferation, and apoptosis, all hallmarks of cancer initiation and progression. Prolonged dysregulation of these pathways has been found to promote uncontrolled cellular growth and malignant transformation, contributing to carcinogenesis and ultimately leading to malignancy. However, the translation of cancer signalling pathways and potential targeted therapies in cancer treatment faces ongoing challenges due to the pleiotropic nature of cancer cells, contributing to resistance and an increased rate of incomplete remission in patients. This review provides analyses of a range of potential anti-cancer compounds in drug repurposing. It unravels the current understanding of the molecular rationale for repurposing these drugs and their potential for targeting key oncogenic signalling pathways.

## 1. Introduction

Cancer is a debilitating disease that poses a major public health issue, accounting for 10 million deaths in 2020 [1]. Cancer progression is characterized by distinctive hallmarks: chronic proliferation, invasion, metastasis, angiogenesis, and evasion of cell death [2,3]. These unique features allow them to stem from normalcy to a neoplastic growth state that is a consequence of both environmental and genetic factors. Environmental factors such as ageing, alcohol consumption, smoking nicotine, exposure to sunlight, ionizing radiation, and diet have been recognized to serve as driving forces in the development and progression of tumourigenesis [4]. However, arguably the most fundamental feature that frequently underlines the initiation and progression of tumour cells is dysregulation of molecular mechanisms that are responsible for the homeostatic control of the growth and division cycle. For instance, instabilities in molecular machinery whereby aberration and mutations of tumour suppressor genes such as *p53* and mismatch DNA repair genes enable the development of oncogenes that confer the advantage of cells to divide uncontrollably and evade tumour suppression [2].

Many of the genes that are commonly mutated in cancers are involved in key cell signalling pathways that are crucial for maintaining homeostatic cellular control [5]. Cell signalling pathways are fundamental processes associated with controlling cell fate, growth, division, and motility. Thus, dysregulation of cell signalling pathways can play a substantial role in tumour cell behaviour. For instance, genes belonging to highly conserved pathways such as the Wingless-related integration site (WNT), Hedgehog (Hh), Hippo, and Notch pathways encode proteins associated with nuclear targets that fuel cancer progression and development [5]. 

Furthermore, it has also been reported that these signalling pathways do not only impact the tumour directly but also exert influence on the surrounding microenvironment, triggering processes such as angiogenesis, migration, and invasion [6]. Therefore, these oncogenic signalling pathways have been extensively explored in cancer research due to their prognostic significance. Understanding these pathways’ architecture provides significant therapeutic opportunities, enabling targeting of specific components associated with tumour cell development [7]. 

### 1.1. Signalling Pathways and Tumourigenesis

Many of the oncogenic mutations that occur in these signalling pathways encode key components that influence the development of cancer-associated processes such as proliferation, growth, survival, and metastasis. Over the past few decades, extensive research has focused on comprehending the modifications in genes and pathways, aiming to identify potential therapeutic interventions and address drug resistance. There are several signalling pathways that have been identified to be frequently mutated in various cancers, including melanoma, breast, pancreatic, uterine, and colon cancers, such as PI3k/Akt, RAS-ERK signalling, MAPK, Wnt/β-catenin, and Hippo pathways [6,8,9,10,11].

Despite an abundance of evidence highlighting changes in various signalling pathways and their involvement in tumourigenesis, this narrative review will focus on the WNT/β-catenin and Hippo signalling pathways and their role in tumourigenesis owing to their intricate nature and implication in tumour initiation, progression, and metastasis [12,13].

#### 1.1.1. The Wnt/β-Catenin Signalling Pathway

The Wnt/β-catenin signalling pathway is a highly conserved evolutionary pathway composed of nineteen richly secreted cysteine glycoproteins that interact with a multitude of subsequent WNT ligands and co-receptors to govern cellular behaviours, essential for such processes as proliferation, differentiation, migration, and maintenance of tissue homeostasis [14]. The Wnt pathway is divided into two major pathways: the canonical (β-catenin-dependent) and the non-canonical (β-catenin-independent) WNT signalling pathways. 

**Figure 1:**
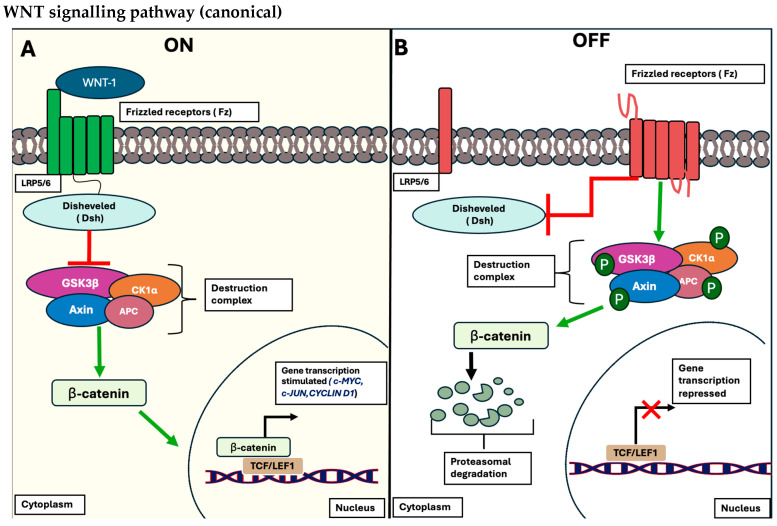
Overview of the canonical pathway (WNT/β-catenin pathway). (**A**) The signalling pathway is considered in the “OFF STATE” when no WNT ligands bind to their respective receptors, LRP5/6, and its co-receptor, Fz receptors. Consequently, no recruitment of the Dsh protein occurs, leading to the phosphorylation of β-catenin by the conserved destruction complex (APC, Axin, PP2A, GSK3β, and CK1α). This phosphorylation triggers the proteasomal degradation of β-catenin, inhibiting its ability to bind to the corresponding transcription factor, TCF/LEF, and regulating the expression of target genes (portrayed as red X). (**B**) The cascade is in the “ON STATE”, where WNT ligands bind to the Fz receptors and their LRP5/6 co-receptors, resulting in the recruitment of Dsh to the plasma membrane, disrupting the β-catenin destruction complex. This disruption allows β-catenin to accumulate in the cytoplasm and translocate into the nucleus, where it interacts with TCF/LEF, initiating the transcriptional expression of target genes such as *C-MYC*, *C-JUN*, and *Cyclin D1* (portrayed as black arrow). This figure is an illustration by Haddad et al. and has not been published elsewhere.

To begin with, the canonical (β-catenin-dependent) pathway is commonly involved in the multistep process responsible for localization, phosphorylation, and degradation of β-catenin, a hallmark protein that serves as a transcriptional factor for a myriad of genes associated with proliferation (c-*MYC*), pluripotency (*Sox2*), and metabolic activity (pyruvate dehydrogenase kinase-1 (*PDK1*)) [15]. When the pathways are activated and considered in the “on-state”, they involve the interactions of specific WNT ligands, such as Wnt-1, to bind to the membrane receptor protein referred to as Frizzled (Fz) and conjugated low-density lipoprotein receptor-related protein (LRP)-5/6 co-receptors on the plasma cell membrane. This subsequently promotes the activation of the intracellular machinery of the WNT pathway, allowing the recruitment of Dishevelled (Dsh) proteins to the plasma membrane and disintegrating the destruction complex; Adenomatous polyposis coli (APC), Axin, protein phosphatase 2A (PP2A), glycogen synthase kinase 3 β (GSK3 β), and casein kinase 1 α (CK1α) [16]. This proteolytic complex is crucial for the proteasomal degradation of phosphorylated β-catenin when the pathway is in the inactivated “off-state”. Thereby, this destruction complex is interrupted, allowing β-catenin to be stabilized in the nucleus and form a complex with both T cell-specific transcriptional factor and lymphoid-enhancing binding factor-1 (TCF/LEF-1) of target genes, *c-MYC*, *c-JUN*, and *Cyclin D1* [17]. A schematic representation is outlined in Figure 1 below. 

**Figure 2:**
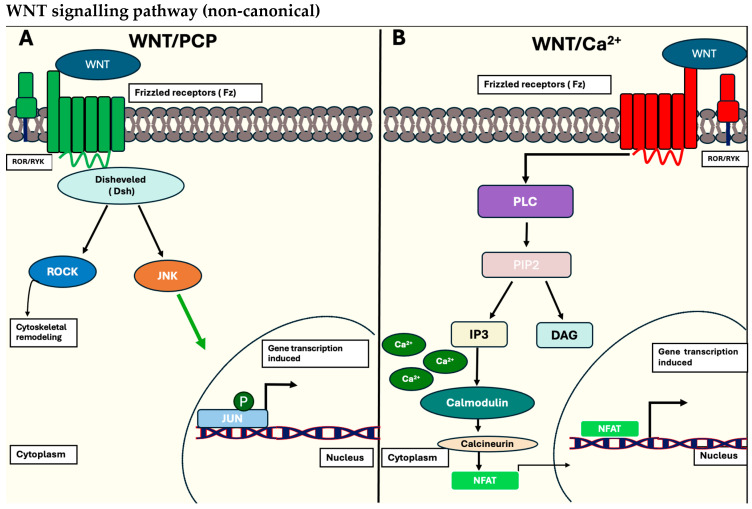
Overview of the non-canonical pathway (β-catenin-independent pathway). (**A**) Activation of the WNT/PCP pathway happens when corresponsive WNT ligands bind to Fz receptors and co-receptors, ROR/RYK, in the plasma membrane. This leads to the recruitment of Dsh proteins that activate either ROCK or JNK, which are responsible for cytoskeletal remodelling or further activation of the JUN transcription factor of target genes. (**B**) WNT ligands bind to Fz receptors and ROR/RYK co-receptors to activate membrane-bound PLC, which allows the hydrolysis of PIP2 to IP3 and DAG. The release of IP3 triggers Ca^2+^ release into the cytoplasm, activating the protein calmodulin. This allows calcineurin expression to be enhanced, resulting in transcription factor NFAT binding to promoter regions and initiating transcription of target genes (indicated as black arrow), associated with ventral-cell fate determination. This figure is an illustration by Haddad et al. and has not been published elsewhere.

On the other hand, the non-canonical pathway (β-catenin-independent pathway) is linked to cellular functions responsible for differentiation, polarity, and migration. This pathway, however, branches into two distinct paths: the planar cell polarity (PCP) pathway and the WNT/calcium (Ca^2+^) pathway. To sum it up, the PCP coordinates the orientation, organization, and motility of the cytoskeleton, while the WNT/Ca^2+^ pathway oversees the secretion of intracellular calcium to coordinate gene expressions related to tissue homeostasis and migration [18]. This is portrayed in Figure 2 below.

#### 1.1.2. The Hippo Signalling Pathway

The Hippo pathway, initially identified in *Drosophila melanogaster*, is an evolutionary pathway consisting of fundamental kinases and regulators. It plays a crucial role in overseeing developmental and regenerative processes essential for cellular growth and development [19]. This pathway comprises core components including Ste20-like protein kinases 1/2 (MST1/2), Warts homolog large tumour suppressor 1/2 (LATS1/2), Mob as tumour suppressors (Mats), WW–homolog protein Salvador (Sav), transcriptional co-activator Yorkie mammalian-homolog yes association protein (YAP), and its PDZ binding motif TAZ (also known as WW domain-containing transcription regulator 1 WWTR1). The central dogma of this pathway revolves around the phosphorylation of YAP/TAZ, which binds to its corresponding transcription factor when dephosphorylated, TEAD (TEA domain-containing protein), leading to the transcription of a diverse array of genes responsible for cell cycle progression and survival [20].

**Figure 3:**
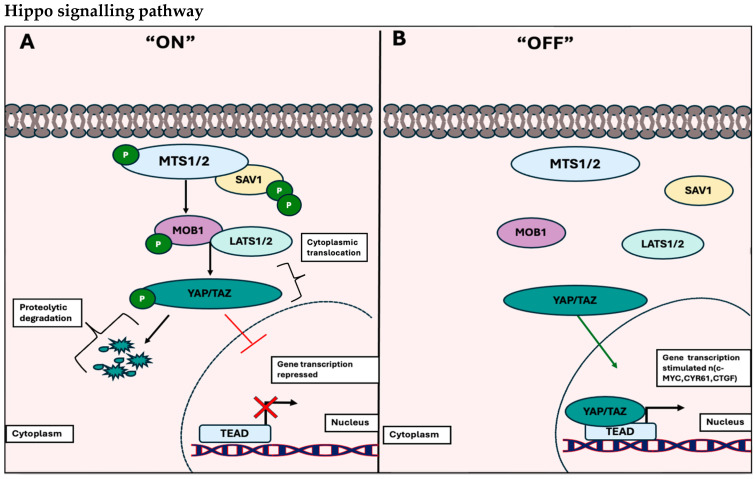
Outline of the Hippo signalling pathway. (**A**) The Hippo signalling pathway is considered in the “ON” state when MTS1/2 is phosphorylated and binds to the respective SAV1 C-domain. This allows a heterodimer to be formed, causing consecutive phosphorylation of MOB1 and LATS1/2 kinases. This results in YAP/TAZ being phosphorylated and recruiting the 14-3-3 protein, causing either cytoplasmic retention or proteolytic degradation. Thus, TEAD ultimately inhibits the transcription of target genes associated with cell proliferation, survival, and migration (perceived in red X). (**B**) The Hippo signalling pathway is in the “OFF” state when no phosphorylation of MTS1/2, SAV1-C domain, MOB1, and LATS1/2 kinases occurs. Therefore, YAP/TAZ is not phosphorylated and is localized to the nucleus, where it binds and forms a complex with TEAD to stimulate transcription of target genes such as: *c-MYC*, *CYR61*, and *CTGF* (perceived as black arrow). This figure is an illustration21 by Haddad et al. and has not been published elsewhere.

The Hippo pathway is also activated either through intrinsic mechanisms such as: cell polarity, adhesion, or cell–cell interactions, or by upstream signals (mechanical or chemical cues). This activation involves the binding of MST1/2 to its respective SAV1 C-domain, forming a crucial heterodimer necessary for the phosphorylation of subsequent proteins, MOB1, and LATS1/2. Subsequently, there is a direct phosphorylation of LATS1/2 by YAP/TAZ, resulting in their cytoplasmic sequestration through mechanistic binding to a site on the 14-3-3 protein or undergoing proteasomal degradation. This is illustrated in Figure 3A below. 

However, when the pathway is deactivated, switching to the “OFF” state, YAP/TAZ undergoes dephosphorylation and is translocated to the nucleus. There, it binds to sequence-specific domains in TEAD. This process mediates the expression of genes associated with inhibiting apoptosis and initiating gene transcriptions essential for tissue growth and cell cycle progression [20]. This is outlined in Figure 3B below.

#### 1.1.3. Deregulation of Cancer Signalling Pathways

Over the last few decades, there has been extensive focus on regulating the Hippo and WNT signalling pathways, given their crucial evolutionary roles in controlling cell fate, survival, and development. Numerous studies have consistently emphasized that abnormalities in gene expression within these pathways can trigger tumourigenesis, often leading to aggressive cancer phenotypes. 

Earlier research on the Hippo signalling pathway has pinpointed core Hippo genes that are frequently altered and associated with tumourigenesis. Wang and her team analysed over 19 core Hippo genes in 9125 patients from the Cancer Genome Atlas (TCGA) database, highlighting somatic copy number alterations and finding mutations present in 33 different cancer types [21]. They reported both mutation and amplification frequency in the following tumour suppressor genes: *TAOK1-3*, *NF2*, *WWC1*, *FRMD6*, *SAV1*, *STK3/4*, *MOB1A/B*, and *LATS1/2*, and oncogenes: *YAP1*, *TAZ*, and *TEAD1-4.* Moreover, they highlighted that among the 33 different cancer types, *NF2*, *LATS2*, *SAV1* mutations, and *YAP* amplification have the highest mutation/amplification frequency in mesothelioma (MESO), kidney renal papillary cell carcinoma (KIRP), and cervical squamous cell carcinoma (CESC) [21]. Further details of Hippo genes and their involvement in tumourigenesis are summarized below in Table 1.

### 1.2. Therapeutic Compounds Targeting the Hippo and WNT Signalling Cascades

The exploration of pharmacological compounds, such as small-molecule inhibitors targeting the Hippo and WNT signalling pathways, is widespread with the aim of effectively modulating or impeding the development, progression, and metastasis of cancer cells. 

SBP-3264, a recently developed MST1/2 inhibitor, aims to impede the tumourigenesis and progression of various haematological malignancies, such as acute myeloid leukaemia (AML) and multiple myeloma (MM). This potent inhibitor stands as a promising hippo target in leukaemia and MM cell lines expressing abnormal serine/threonine-protein kinases 3 (STK3/MST2) and 4 (STK4/MST1) expressions [26]. Consequently, it has been observed that SBP-3264 influences the proliferation and survival of AML and MM cells by inducing apoptosis through the activation of the tumour suppressor gene, TP53 [27]. This effect is believed to be attributed to SBP-3264’s capability to restore low YAP1 levels, a crucial tumour suppressor in haematological malignancies. Thus, inhibiting STK3/4 is hypothesized to reactivate YAP1 levels, inducing DNA-induced damage in AML and MM cells [27]. Additionally, another MST1/2 inhibitor, XMU-MP-1, has also emerged as a potential target for various pathologies, including prostate and breast cancers. This is due to studies that identified STK3 expression as being amplified in metastatic prostate cancer and associated with poor outcomes in patients [28]. In line with this evidence, Schirmer and her team observed that XMU-MP-1 treatment significantly slowed the proliferation and invasive properties of prostate cancer cells. Furthermore, when treating the BC cell line, LNCaP, with XMU-MP-1, a similar reduction in YAP1 phosphorylation was reported, leading to decreased cell proliferation. 

Similarly, recent research has emphasized the development of molecular inhibitors targeting YAP/TEAD complexes as a promising strategy to selectively address cancer cells exhibiting elevated YAP and TAZ expressions. An example includes the exploration of verteporfin, a benzoporphyrin photosensitizer derivative initially used for neovascular macular degeneration and to hinder cell proliferation in endometrial cancer (EC). Wei and his colleagues found that verteporfin suppresses proliferation, migration, and invasion of EC cells that are resistant to conventional progestin-based therapy [29]. Another YAP-transcriptional suppressor, Vestigial-like-4 (VGLL-4), has also recently emerged as a novel tumour suppressor in colon cancer by regulating both the YAP/TAZ and β-catenin-TCF4 complex [30]. This was highlighted by Jiao and his team in a study published in 2017 that reported VGLL4 peptide mimics suppress CRC tumour growth by interfering with the transactivation of YAP/TAZ and the β-catenin/TCF4 complex, thereby preventing the constitutive transcription of WNT and Hippo oncogenic target genes. Their study elucidates VGLL4 as a promising therapeutic in co-regulating gastric tumours that express abnormal levels of WNT and Hippo gene expressions [31].

The WNT/β-catenin signalling pathway is recognized as an intricate network consisting of diverse extracellular receptors, co-receptors, and ligands crucial for inducing downstream activation of β-catenin nuclear signalling cascades. Consequently, there has been a recent surge in strategies to develop innovative therapeutic agents capable of impeding tumour initiation and progression by disrupting novel interactions between extracellular receptors and ligands. OMP-18R5 (vantictumab) is a monoclonal antibody aimed at inhibiting Fzd receptors and blocking the activation of the canonical WNT signalling in multiple tumour types, such as in CRC, BC, and pancreatic cancer (PC). In a study carried out by Gurney and his team, they showed that CRC, BC, and PC treated with OMP-18R5 lead to a reduction in tumour proliferation, cell frequency, and growth. Additionally, they reported significant inhibition of several WNT target genes, such as *SOX1* and *SOX2*, when treated synergistically with chemotherapy like gemcitabine [32]. 

Another therapeutic agent that has been observed to successfully antagonize the WNT/β-catenin pathway is OMP-54F28. OMP-54F28 (ipafricept), a Fzd8 protein fused with immunoglobulin IgG1, has been shown to inhibit tumour proliferation by binding to Fzd receptors on solid tumours. In a phase 1 trial, OMP-54F28 was observed to significantly inhibit the proliferation and growth of solid tumours such as prostate, breast, and non-small lung cancers. After showing promising results in solid tumours, a phase 1a trial was conducted that involved a first-in human study. It was reported that the drug was tolerable in patients and influenced prolonged survival. Due to this, the drug was further studied in three phase 1b trials in which the drug was combined with chemotherapeutic agents, nanoparticle albumin-bound pactlitaxel and gemcitabine. This showed a successful response rate with prolonged survival and delayed tumour recurrence and regrowth in patients [33].

### 1.3. Challenges of Targeted Therapy in Cancer 

It is vital to recognize that despite the success demonstrated by current WNT- and Hippo-mediated therapies in inhibiting oncogenic signalling pathways driving tumour progression, they have faced specific challenges that limit their application as anticancer agents in combating tumours. These challenges arise from our evolving understanding of cancer cells, their microenvironment, and tumour heterogeneity, which collectively contribute to the development of tumour cell clones that become resilient to treatment due to selective pressure [34]. Furthermore, the translation of potential targeted therapies may be hindered by secondary mutations triggered by conventional treatments, causing the activation of oncogenic rearrangements in target genes. It is also crucial to underscore the clinical outcome of targeted therapies for patients. While targeted therapies are generally regarded as safe and non-toxic because they selectively target specific cancer cells, they can also come with off-target effects, which means they might inhibit proteins other than the intended target, or on-target effects, where they inhibit the targeted protein in healthy cells [35].

Taking all these factors into consideration, there has been a shift in the approach to using targeted therapy, focusing on repurposing older drugs that have already been approved and proven safe, effective, and well-tolerated in patients. This signifies a novel alternative approach called drug repurposing, which has been demonstrated as a promising field in the fight against cancer. 

### 1.4. Drug Repurposing 

Drug repurposing, also called ‘drug repositioning’, is the process of using existing medications or previously approved drugs for alternative purposes contrary to their initial indication. This approach accelerates the process of drug discovery by finding a novel indication for a pre-existing drug. The process of drug development is a time-consuming and costly process with a high risk of failure, whereas with drug repurposing, time is significantly reduced as target discovery, lead discovery, and optimization required in de novo drug development are circumvented [36]. This strategy requires no alterations to the chemical structure of the drug but rather discovers alternative indications based on the approved biological properties or leverages its side effects for beneficial purposes. In addition, it lowers the risk of failure with a high success rate and overall minimizes the cost of drug discovery [37].

Drug repurposing has become increasingly feasible given the capacity to characterize diseases based on their molecular profiles, which encompass genes, biomarkers, signalling pathways, and environmental factors [38]. Computational methodologies such as data mining can then be used to assess similarity between diseases that already exhibit a range of shared molecular features [39,40]. For instance, Parkinson’s disease and Alzheimer’s disease have 48 genes and four signalling pathways in common. The identification of common protein targets among different diseases hints at the possibility that a particular drug could be effective in treating both conditions [37]. 

Recently, there has been a growing focus on drug repurposing as an emerging therapeutic avenue for the treatment of cancer. This approach is particularly beneficial, with one of its key advantages lying in the significantly reduced time frame compared to the development of entirely new drugs. This process consists of essential steps to assess the repurposing potential of a particular drug. This is not limited to only pre-clinical methodologies such as in silico, in vitro, or in vivo studies but also incorporates clinical observations and epidemiological studies [41]. Therefore, it is possible to identify promising drugs to repurpose for cancer therapy by studying the targeted molecular pathways and their anti-neoplastic effects [10]. 

#### Stages of Drug Repurposing

**Figure 4:**
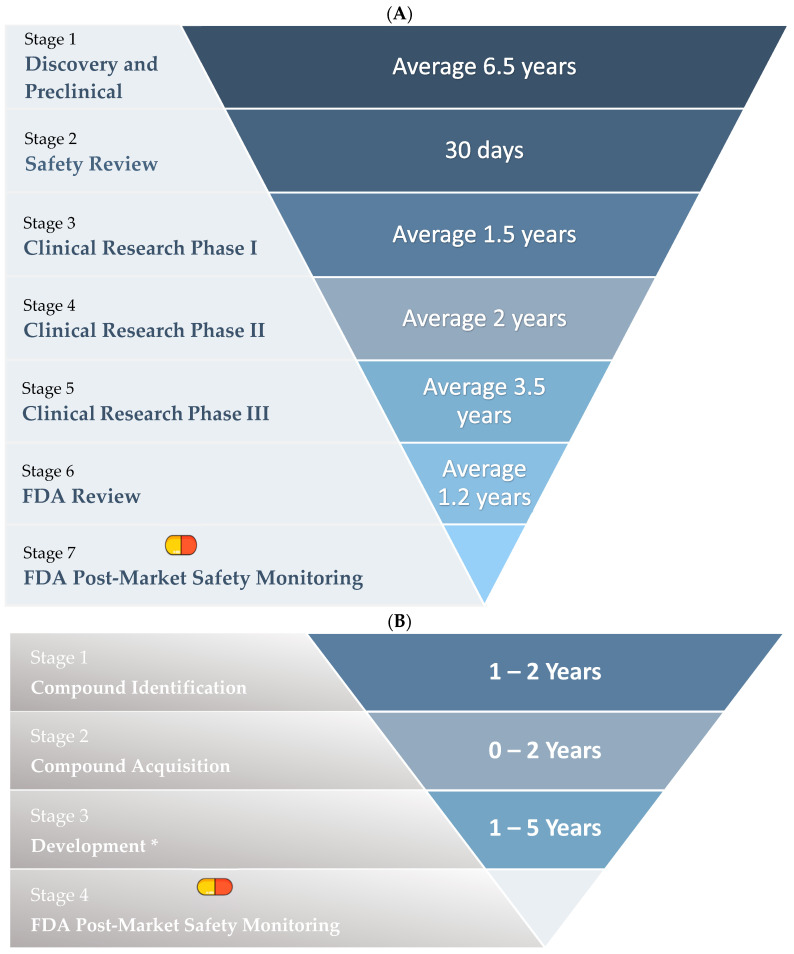
(**A**): **Drug discovery and development funnel.** The diagram illustrates the individual steps involved in the development of a new drug in comparison to drug repurposing. (**B**): **Drug repurposing funnel.** The diagram highlights the fewer steps involved in drug repurposing when compared to conventional drug development. * The development stage includes preclinical, phase 1, and phase 2 drug research. This figure is an illustration by Haddad et al. and has not been published elsewhere.

The conventional drug development process comprises five distinct phases: discovery and preclinical, safety review, clinical research, FDA review, and FDA post-market safety monitoring. This comprehensive process spans approximately 14.5 years (as shown in Figure 4A). In contrast, drug repurposing streamlines the procedure by omitting one stage. It involves compound identification, compound acquisition, development (including preclinical, phase 1, and phase 2 drug research), and FDA post-market safety monitoring, thereby completing the process in a condensed timeframe of just over 3 years (as shown in Figure 4B) [40]. 

### 1.5. Examples of Repurposed Drugs

The first drug to be successfully repurposed was acetylsalicylic acid. It was originally marketed by Bayer in 1899 as an analgesic, then repurposed in the 1980s as an antiplatelet aggregation drug [37]. 

Structure-based drug repurposing is significant due to its capacity for multi-protein binding. When medications exhibit affinities for proteins beyond their primary therapeutic agents, it is termed off-target interaction. For instance, sildenafil, originally indicated for angina and hypertension, displayed interactions with phosphodiesterase (PDE5), leading to its repurposing as Viagra^®^ by Pfizer for erectile dysfunction [42,43]. Similarly, thalidomide, originally prescribed for motion sickness in pregnant women, was prohibited due to its association with severe birth defects. Nonetheless, it was subsequently reapproved for therapeutic use in the treatment of multiple myeloma, along with additional novel indications [42].

Current research has discovered that multiple drug classes with indications to different diseases can be repurposed for the treatment of cancer. Essentially, a few anti-psychotic, anti-malarial, anti-diabetic, and anti-viral drugs can be repurposed for the treatment of specific cancers [41], as shown in Table 2.

## 2. Repurposing Psychotropic Medications as Potential Therapeutic Drugs for Cancer

Cancer is a heterogeneous disease known for its ability to develop resistance to various standard chemotherapy treatments, leading to reduced treatment efficacy and increased rates of relapse among patients. Consequently, research efforts have been directed towards repurposing drugs with the aim of acting as chemosensitizers and potentially exerting anticancer effects. Significant strides have been made in cancer biology by exploring psychotropic medication as a promising new strategy against cancer. This interest has been spurred by growing epidemiological evidence indicating that patients receiving prolonged psychotropic medications have lower cancer incidence rates compared to the general population [46]. Therefore, considering this data, pre-clinical studies have taken psychotropic medications forward and investigated the potential anti-tumoral activities of these medications. 

Fortunately, experimental evidence has suggested that a class of psychotropic medications known as selective serotonin reuptake inhibitors (SSRIs) exhibit anti-cancer properties. When combined with chemotherapy, SSRIs can effectively reverse drug resistance in cancer cells at safe and lower doses, thus highlighting their unique role as repurposed chemosensitizers [47]. SSRIs are antidepressant medications that are widely implemented in individuals suffering from depression, anxiety, autism, eating disorders, and nociceptive pain. 

SSRIs are considered unique first-line treatments due to their tolerability and safety compared to older-generation drugs like tricyclic antidepressants (TCAs). TCAs are often associated with adverse side effects such as cardiac block, arrhythmia, and seizures [48]. This is primarily due to SSRIs’ primary mechanism of action in blocking the reabsorption of serotonin in the presynaptic neuron by inhibiting the respective serotonin receptor, 5-HTP (SERT), causing an increase in serotonin levels in the synapses and post-synaptic membrane [49]. Therefore, SSRI’s have been widely considered in cancer biology due to their clinical benefits and experimental evidence that highlighted their role in modulating cancer cell behaviours. 

### 2.1. Sertraline 

Among the SSRIs, sertraline, a first-line antidepressant agent, has been widely considered in clinical studies due to encouraging results portraying its ability to elicit anti-tumour properties in various cancers such as colorectal, breast, ovarian, and prostate cancers [50]. These findings underscore the role of sertraline in modifying serotonin levels within the tumour microenvironment, effectively reducing the viability, growth, and proliferation of aggressive cancer cell lines [51]. 

Sertraline has been perceived to elicit an anti-tumour effect by reversing multidrug resistance mechanisms in numerous malignant tumours. For instance, a study investigated by Gil-Ad and his team evaluated the effect of sertraline on both human colon carcinoma (HT29) and MRD colorectal cancer (LS1034) cell lines [52]. They reported sertraline to reduce cell viability, induce apoptotic cell death, increase activity of caspaspe-3, and increase expression of antiapoptotic protooncogenes (*p53*, *Bcl-2*, and *c-Jun*) in both HT29 and LS1034 cell lines when treated with 10 and 20 µM of sertraline. Therefore, their results tend to support the use of sertraline as an anti-cancer drug due to their superiority in inducing cytotoxicity in both carcinoma and multidrug resistance cell lines. Furthermore, sertraline has been shown to be an effective chemosensitizer when combined with conventional chemotherapeutic drugs such as cisplatin and doxorubicin. As such, a study highlighted by Baldisseri and his team investigated the effect of sertraline on translational controlled tumour protein (TCTP) on different breast cancer cell lines (PMC 42, MCF7, SKBR3, MDA-MB-231, and MDA-MB-4360) [53]. TCTP is a protein and histamine-releasing factor conserved in eukaryotic stem cells, often associated with contributing to the prosurvival of cancer cells and promoting their ability to confer malignant phenotypes and resist apoptosis induced by chemotherapy [54]. Baldisseri and the team reported that cell lines with overexpressed TCTP levels, MDA-MB-231 and MDA-MB-4360, were sensitized with sertraline before the addition of chemotherapeutic agents (cisplatin and doxorubicin), resulting in a more effective cytotoxic effect than when treated alone. They observed a decrease in cell viability of 55% and 64%, compared to 25% and 45% when treated with cisplatin and doxorubicin alone. Thus, their data suggest that the combination of sertraline with chemotherapeutic agents can enhance patient response, promote remission, and potentially reduce the risk of relapse [53]. 

Although numerous studies have underscored the antitumour role of sertraline, the exact molecular mechanism behind its effect remains unknown. However, recent research indicates an association between serotonin and YAP signalling exists. It is suggested that elevated levels of intercellular serotonin may promote tumour growth and sustain cell proliferation by enhancing the transduction of YAP signalling. This aspect was investigated in a study by Yu and his team, which explored the impact of serotonin and different SSRIs (citalopram and fluvoxamine) on various colon cancer cell lines (SW480 and SW1116) [55]. Initially, they found that serotonin stimulates cell proliferation through 5-HT signals, supporting the idea of serotonin as a mitogenic factor that facilitates colon cell proliferation and development by enhancing YAP nuclear translocation and transcriptional activity. Additionally, they observed that treatment with citalopram and fluvoxamine regulates YAP expression, suggesting that inhibiting serotonin receptors and transporters reduces intracellular serotonin levels, thereby modulating YAP expression in CRC [55]. As a result, this investigation offers supporting evidence for the prospective application of SSRIs, like sertraline, to suppress serotonin receptor signalling. This signalling pathway is intricately connected with core proteins in intercellular signal transduction, such as the YAP protein in the hippo signalling pathway. This inhibition aims to impede the development, progression, and growth of colorectal cancer. 

### 2.2. Fluoxetine 

Fluoxetine is a widely prescribed SSRI used for treating depression in cancer patients. However, like sertraline, its utilization has been considered a potential antitumour therapeutic due to its inhibitory effect on carcinogenesis. Numerous studies have highlighted fluoxetine’s effectiveness in regulating cancer cell behaviour, either by directly inhibiting tumour growth or modulating the neoplastic tumour microenvironment by modifying T-cell immunity. 

For instance, in a study conducted by Frick et al. [56], chronic administration of fluoxetine demonstrated an inhibitory effect on lymphocytes derived from aggressive T-cell lymphoma. The findings suggested that this effect was attributed to fluoxetine’s role in enhancing the immune response rather than directly targeting tumour cells. The study revealed a significant increase in the population of CD4+ T-helper and CD8+ T-cytotoxic lymphocytes, along with elevated production of anti-cancer cytokines (IFN-γ and TNF-α). Consequently, the study provides supporting evidence for fluoxetine’s crucial role in targeting Th1-type immunity by augmenting T-cell lymphocytes and promoting anti-tumour cytokine production and response. 

Furthermore, fluoxetine’s anti-tumour mechanisms have also been extensively investigated in recent studies to show a direct inhibition of the proliferation and growth of tumour cells. This is perceived in a study by Kanner and his team [57] that evaluated the effects of fluoxetine in vivo on colon tumour cells in rats by assessing their impact on proliferation, angiogenic activity, and serotonin metabolism. They reported fluoxetine to exhibit oncostatic properties by showcasing reduced crypt proliferation and suppressing the development of dysplastic aberrant crypt foci (ACF). Additionally, their study demonstrated fluoxetine’s ability to inhibit tumour microvascular formation around the crypts by reducing vascular epidermal growth factor (VEGF) secretion and constricting tumour arterioles, leading to suppressed tumour proliferation and growth [57]. 

Hence, our comprehension of fluoxetine’s potential anti-tumour role promotes confidence in its application as a novel approach for cancer patients. However, like sertraline, the precise molecular mechanism underlying its antitumour behaviour remains elusive. Nevertheless, there is promising data suggesting that fluoxetine can influence the development and progression of tumour cells by targeting oncogenic signalling pathways, notably the WNT canonical pathway. 

This was demonstrated in the study conducted by Warkus and Marikawa [58], which investigated the impact of fluoxetine on down-regulating relative pluripotency genes critical for embryo patterning and morphogenesis that are known to contribute to cancer initiation and progression when aberrant. They reported that fluoxetine at concentrations of 6 µM or higher tended to diminish relative WNT genes such as *Sox2*, an SRY-box transcription factor, and ligands like *Wnt3a*, thereby inhibiting the WNT canonical pathway and altering embryonic morphogenesis. Thus, the reduction in embryoid morphogenesis suggests fluoxetine’s role in diminishing cell proliferation, reducing cell growth, and impacting the morphogenesis of embryonic cells. These findings suggest the potential of fluoxetine as a WNT inhibitor to alter the proliferation and viability of cancer cells, encouraging its use as an anti-cancer treatment. 

### 2.3. Repurposing Antimalarials as Potential Therapeutic Drugs for Cancer 

#### Artemisinin/Artesunate 

Commonly used antimalarial drugs, artemisinin (ARTM) and its semisynthetic derivate, artesunate (ART), have been widely researched for their anticancer effects against numerous cancers. Deriving from extracts of sweet wormwood, Artemisia annua, is a cost-effective alternative to be repurposed for the treatment of cancer [59]. Amongst the wide range of antimalarial properties, artemisinin and artesunate consist of a variety of antitumour properties, targeting the most common tumour cell lines, including cervical, lung, colon, breast, pancreatic cancers, and even leukaemia [60]. For instance, studies have identified that ART diminishes the proliferation of colorectal cancer cells and increases apoptosis by inhibiting the overactive Wnt/β-catenin pathway commonly indicated in colorectal cancer [61]. Moreover, through RT-PCR and immunofluorescence analysis on Wnt/β-catenin target genes, it was discovered that β-catenin was translocated from the nucleus by ART to minimize transcription [61]. Additionally, following an ELISA analysis, ART’s inhibitory effects on vascular endothelial growth factor expression (VEGF) receptor KDR/flk-1 in tumour cells in chronic myeloid leukaemia (K526 cells) were discovered. According to the results, a concentration as low as 2 μmol/L of ART could lower the secreted levels of VEGF [62]. 

Studies suggest that artemisinin presents radiosensitising effects on cervical cancer following a clonogenic survival assay, flow cytometry, and western blot analysis [63,64]. The cell viability of Hela, SiHa, and fibroblast cells was analysed post-treatment with different concentrations of ARTM and radiation. The results indicated higher cytotoxicity in cervical cell lines compared to fibroblasts [63]. Further analysis using clonogenic assays and apoptosis revealed higher sensitization of HeLa cells treated with ARTM to the cytotoxicity caused by radiation. Radiation led to the activation of Wee 1 and a reduction in the expression of cyclin B1, which was reinstated to the control level by ARTM as it evaded the G2 blockade induced by radiation. Thus, we conclude that ARTM is a potential radiosensitizer via the regulation of Wee 1 and cyclin B1 expression [63]. Similarly, nude mice inoculated with HeLa and SiHa cells were treated with ART to assess the effect of radiosensitivity using flow cytometry. Analysis indicated no radiosensitivity of SiHa but an enhanced effect on HeLa cells instead, concluding that ART’s ability to promote radiosensitivity and evasion of G2 checkpoint control is similar to ARTM in HeLa cells [64]. 

Additionally, Li et al.’s study demonstrates that upon administration of ARTM, signal transduction of Hippo-YAP is noticeably inactivated in hepatocellular carcinoma cells. This was supported by a Western blot analysis, which revealed decreased nuclear YAP and increased p-YAP Ser 127 in ARTM treated HCC whole cells and cytoplasm [65]. 

## 3. Drug Repurposing in Targeting Alternative Cancer-Associated Signalling Pathways

The popularity of drug repurposing has also been widely considered in targeting alterative signalling pathways that contribute to the progression of cancer. Cancer does not only stem from aberrant regulation of mitogenic signalling pathways, but there is also evidence that has identified inflammatory processes to significantly contribute to the progression of tumours. This is due to our current understanding of the inflammatory mechanisms that have been perceived to influence cancer progression due to supporting evidence highlighting the presence of inflammatory cells in the tumour microenvironment [66]. 

Inflammation is composed of multifactorial networks of **cellular components**: platelets, leukocytes (neutrophils, monocytes, and eosinophils), tissue mast cells, and **pro-inflammatory and chemotactic cytokines** (tumour necrosis factor-α (TNF-α) transforming growth factor-β1 (TGF-β1), platelet-derived growth factor (PDGF), interleukins-1, 1α, -1β, and -4 (IL-1α, -1β, and -4)). These elements play a vital role in response to tissue injury and wound healing as they stimulate and immobilize leukocytes, secrete growth factors, accelerate coagulation, and undergo tissue repair via re-epithelialisation [67].

The activation of key cellular processes that occur during wound healing and inflammation is crucial to be tightly regulated, as their dysregulation significantly promotes the progression and dissemination of tumours. The **nuclear factor-κB (NF-κB)** signalling pathway is a well-recognized family of inducible transcription factors that modulate a diverse array of genes associated with inflammatory responses. They regulate the activation, differentiation, and survival of pro-inflammatory genes, cytokines, chemokines, and immune cells [68]. Therefore, it is not surprising that deregulation of the **NF-κB** pathway serves as a significant hallmark of cancer. Taking this into consideration, repurposed drugs are being explored to modulate inflammation-associated processes that promote tumour malignancies.

For instance, sildenafil, commercially known asViagra^®^, is a potent phosphodiesterase-5 (PDE5) inhibitor that is used for the treatment of erectile dysfunction [69]. However, it has been recently repurposed as an anti-cancer drug due to an investigation regarding its role as a targeted inhibitor of the NF-κB pathway. This is portrayed in a recent study that demonstrated co-treatment of sildenafil with chemotherapy, doxorubicin, to enhance cell cytotoxicity by inhibiting NF-κB in prostate cancer cells [70]. A recent study reported that sildenafil inhibits the NF-κB transcription factor from binding to the promoter region of the protein-tyrosine phosphatase FAP-1 gene, which is associated with apoptotic resistance in various malignancies, including prostate cancer. Treatment of DU145 prostate cancer cells with sildenafil in this study resulted in reduced activation of NF-κB and consequent downregulation of the FAP-1 protein. This inhibited anti-apoptotic proteins such as C-FLIP from blocking caspase-8 and numerous pro-apoptotic proteins (Bid, Bad, and Bax), thereby stimulating apoptosis and reducing the cells’ resistance to Fas-mediated death [70].

## 4. Conclusions

In conclusion, this review highlights the comprehensive approach of utilising drug repurposing as a novel solution for addressing the expanding issues of standard cancer treatments. This is due to several obstacles to current cancer treatments that have been shown to ineffectively inhibit tumour growth or induce cell death, resulting in increased patient relapse and failure to achieve remission, as well as tumour reoccurrence and metastasis.

Therefore, repurposing already-approved drugs instead of de novo drug development has provided substantial evidence to overcome the challenges associated with cancer treatment, including prolonged development timelines, escalating costs, low approval rates, and predominantly tackling the heterogonic nature of tumours and their mechanism of drug resistance. Thus, recent approaches in cancer research have shifted to utilising existing drugs that have demonstrated evidence of successfully targeting tumours with altered oncogenic signalling pathways, particularly those contributing to the mitogenic nature of cancer cells.

Recently, the highly evolutionary conserved Hippo and Wnt/β-catenin signalling pathways have gained considerable attention in cancer medicine. This is due to studies that have shown dysregulation of these pathways to be associated with tumour formation and progression in many epithelial and solid tumours, including colorectal, breast, prostate, thyroid, melanoma, cervical, and lung cancers. As a result, recent studies have urged the importance of repositioning drugs that hold promise as potential Hippo and Wnt/β-catenin therapeutic targets.

Thus, recent studies have suggested the potential use of psychotropic medications or antimalarial agents as effective cancer therapies. This is attributed to their ability to suppress tumour growth, proliferation, and development by interacting with key components of the intricate Hippo and Wnt/β-catenin pathways. However, it is important to note that, as of current knowledge, there is no evidence supporting these drugs as approved anticancer agents through their direct mechanistic role as Hippo and Wnt/β-catenin modulators or inhibitors.

Nonetheless, this review provides valuable molecular insights into the repositioning of drugs showing promise in targeting tumours with dysregulated Hippo and Wnt/β-catenin pathways, while also highlighting the importance of drug repurposing in alternative signalling pathways that are associated with tumourigenesis such as the NF-κB pathway. These findings provide a pragmatic approach for pharmaceutical researchers to investigate the use of non-oncology drugs as effective treatments for cancer, with the aim of ultimately improving patient outcomes.

## Figures and Tables

**Table 1:** Various modified Hippo genes and their role in driving tumourigenesis in different cancer cells.

Cancer Type	Hippo Genes	Type of Alteration	Role in Tumourigenesis	References
Breast Cancer (triple- negative subtype)	*YAP/TAZ*	Amplification	TAZ expression induces metastatic and aggressive-like properties in breast cancer stem cells.	[22]
Uveal Melanoma	*YAP*	Amplification	*YAP-TEAD* activation leads to transcription of canonical target genes (*CTGF*, *CYR61*, and *AMOTL2*) and *c*-*MYC* hyperactivation.	[23]
Colon Cancer	*YAP1/TAZ*	Amplification	*YAP1* expression is associated with enhanced transcription of target genes associated with colon cancer progression and poor prognosis.	[24]
Glioblastoma	*YAP/TAZ*	Overexpressed	YAP/TAZ prevents glioblastoma–stem cells from differentiating and mediates cell cycle progression in the gliomaspheres.	[25]

**Table 2:** Widely repurposed drugs for different cancers. A summary of the different drugs repurposed for the treatment of various cancers [44,45].

Cancer Type	Drug Name	Original Indication
General cancers	*Gemcitabine*	Antiviral
Breast cancer	*Raloxifene*	Osteoporosis
Breast, colorectal, endometrial, and prostate cancers	*Metformin*	Diabetes
General cancers	*Orlistat*	Obesity
General cancers	*Itraconazole*	Fungal infection
Multiple myeloma, neuroblastoma, and leukaemia	*Flubendazole*	Anthelmintic drug
Multiple myeloma	*Thalidomide*	Morning sickness
Colon cancer	*Azithromycin*	Antibacterial drug
Bone and prostate cancers	*Doxycycline*	
General cancers	*Itraconazole*	
Lymphoma and leukemic cells	*Griseofulvin*	Antifungal
Glioblastoma	*Clotrimazole*	
Breast and colorectal cancer	*Ciclopirox*	
Colorectal cancer	*Aspirin*	
General cancers	*Etodolac*	NSAIDs/Anti-inflammatory
General cancers	*Etoricoxib*	
General cancers	*Celecoxib*	
Breast cancer and glioblastoma	*Hydroxychloroquine*	Antimalarial drugs
Lung, breast, and colon cancers	*Atovaquone*	

## Data Availability

The study did not report any new results or data.
